# Dual Acting Polymeric Nano-Aggregates for Liver Cancer Therapy

**DOI:** 10.3390/pharmaceutics10020063

**Published:** 2018-05-26

**Authors:** Wejdan Al-Shakarchi, Ali Alsuraifi, Anthony Curtis, Clare Hoskins

**Affiliations:** 1 Institute of Science and Technology in Medicine, School of Pharmacy, Keele University, Keele ST5 5BG, UK; w.n.s.al-shakarchi@keele.ac.uk (W.A.-S.); a.t.y.alsuraifi@keele.ac.uk (A.A); a.d.m.curtis@keele.ac.uk (A.C.); 2 College of Pharmacy, Mosul University, Mosul 41002, Iraq; 3 College of Dentistry, University of Basrah, Basrah 61004, Iraq

**Keywords:** combination therapy, drug delivery, liver cancer, paclitaxel, hybrid nanoparticles

## Abstract

Liver cancer treatments are often hindered by poor drug physicochemical properties, hence there is a need for improvement in order to increase patient survival and outlook. Combination therapies have been studied in order to evaluate whether increased overall efficacy can be achieved. This study reports the combined treatment of liver cancer cells with a combination treatment of chemotherapeutic agent paclitaxel and pro-apoptotic protein cytochrome C. In order to administer both agents in a single formulation, a poly(allylamine)-based amphiphile has been fabricated with the incorporation of a hybrid iron oxide-gold nanoparticle into its structure. Here, the insoluble paclitaxel becomes incorporated into the hydrophobic core of the self-assemblies formed in an aqueous environment (256 nm), while the cytochrome C attaches irreversibly onto the hybrid nanoparticle surface via gold-thiol dative covalent binding. The self-assemblies were capable of solubilising up to 0.698 mg/mL of paclitaxel (700-fold improvement) with 0.012 mg/mL of cytochrome C also attached onto the hybrid iron oxide-gold nanoparticles (HNPs) within the hydrophobic core. The formulation was tested on a panel of liver cancer cells and cytotoxicity was measured. The findings suggested that indeed a significant improvement in combined therapy (33-fold) was observed when compared with free drug, which was double the enhancement observed after polymer encapsulation without the cytochrome C in hepatocellular carcinoma (Huh-7D12) cells. Most excitingly, the polymeric nanoparticles did result in improved cellular toxicity in human endothelian liver cancer (SK-hep1) cells, which proved completely resistant to the free drug.

## 1. Introduction

Liver cancer is a devastating disease with around 5700 patients newly diagnosed in the United Kingdom alone each year [1]. Current treatment regimens often are not effective because of the late stage of diagnosis and the inability to deliver the correct therapeutic dose to the liver [2]. Over the past few years, more effort has been concentrated in the development of multifunctional [3,4,5] or combination therapy [6,7,8] in order to improve existing anticancer treatments. This strategy has previously identified that combination therapy can result in synergy and overall can improve efficacy of therapies, as well as reduced drug resistance. These studies often focus on the use of two or more chemotherapy agents which, however, often results in a costly approach that may not be feasible for the market [9]. In order to overcome these challenges, the use of proteins that can be used to trigger or program cell death has been explored as an alternative to existing drug entities. Nanotechnologies have been extensively shown to accumulate within the liver after systemic circulation [10,11,12]. This accumulation can be exploited for liver cancers in order to deliver the therapeutic payload in a site specific manner.

Previously, we demonstrated that by attaching pro-apoptotic protein cytochrome C onto the surface of hybrid iron oxide-gold nanoparticles (HNPs) and delivering these particulate systems to liver cancer cells in combination with chemotherapy agents, a synergistic effect resulted in increased cellular mortality [13,14]. In this study, we aim to prepare a single formulation combining both cytochrome C and chemotherapy treatment. Our proof of concept studies showed that combined delivery of HNP surface functionalised with cytochrome C (HNP-c) and paclitaxel (PTX), resulted in the most notable effects across hepatocellular carcinoma cell line (HepG2), hepatocellular carcinoma cell line (Huh-7D12), and human endothelial liver cancer (SK-hep-1) cell lines [14]. PTX is a member of the taxane anti-microtubule drug family, which has exhibited a potent effect in cancer treatment by targeting the β-tubulin subunit of the microtubule [15]. Here, the PTX binds the tubulin parallel to the microtubule and increases the polymerization processes. Consequently, conformational changes at the M-loop part of the β-tubulin and a stable interaction between the proto-filament in the lateral level are observed. However, because of its poor aqueous solubility, clinical use of PTX is often hindered [16]. As such, formulations of PTX using amphiphilic polymers have been heavily reported in the literature [17,18,19,20], with some nanoparticle formulations now available in the clinic for cancer therapy [21].

Unfortunately, it is not possible to directly conjugate PTX onto the gold surface of the HNPs to co-deliver the drug with cytochrome C. This is because of the fact that there is no obvious mechanism, such as the presence of thiol or Van der Waal’s interactions, required for direct binding. Additionally, the highly hydrophobic nature of the PTX molecule would result in the HNPs precipitating out of solution. Hence, a more sophisticated macromolecular system is required if the HNP-c and PTX are to be co-delivered in a single platform.

**Figure 1:**
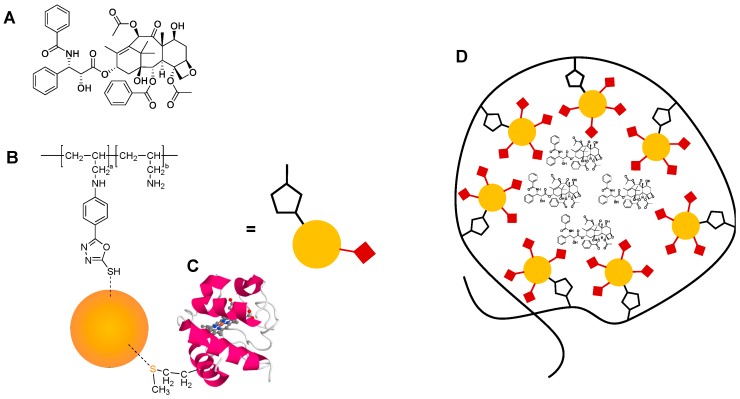
Chemical structure of (**A**) paclitaxel and (**B**) poly(allylamine)-oxadiazole (PAA-Ox5)-hybrid iron oxide-gold nanoparticle (HNP), and molecular structure of (**C**) cytochrome C and (**D**) novel formulation.

In this work, we report the use of an amphiphilic polymer with HNPs incorporated into its intrinsic structure for combined delivery of cytochrome C and PTX (Figure 1). The amphiphilic polymer used is a graft polymer based on an aqueous poly(allylamine) with hydrophobic 5-(4-chlorophenyl)-1,3,4-oxadiazole-2-thiol (oxadiazole) pendants grafted in a 5% molar ratio onto its homopolymer backbone, forming poly(allylamine)-oxadiazole (PAA-Ox5). The thiol residues in the oxadiazole pendants are capable of forming irreversible dative covalent binding with the gold surface of HNPs. Once in aqueous environments, these amphiphilic structures spontaneously aggregate into core-shell aggregates because of a reduction in Gibbs’ free energy. It is this spontaneous aggregation that allows for hydrophobic drug incorporation and solubilisation within the core.

This PAA-Ox5 construct has previously been reported as a magnetomicelle with the potential for drug solubilisation [22]. Unfortunately, the cytochrome C itself is freely water soluble and, hence, has no thermodynamic desire to become encapsulated inside the core of the self-assemblies formed. Hence, in order to produce a single combined therapy platform, HNPs are incorporated into the intrinsic polymer structure, allowing for both drug solubilisation of the hydrophobic PTX and the ability to carry cytochrome C as a cargo attached to the HNP surface, which sits inside the core of the nano-aggregates formed. As far as we are aware, this is the first in kind magnetomicelle used for combined therapy of cytochrome C and chemotherapies for liver cancer treatment.

## 2. Materials and Methods

All chemicals, unless otherwise stated, were purchased from Sigma-Aldrich (Gillingham, UK), all solvents and cell culture consumables were purchased from Fisher Scientific (Loughborough, UK). Hepatocellular carcinoma cell line (HepG-2) were purchased from American Type Culture Collection (ATCC, Manassas, VA, USA) (ATCC^®^ HB8065™), SK-hep-1 was from ATCC (ATCC^®^ HTB-52™), Huh-7D12 cell line was from ECACC (European Collection of Authenticated Cell Cultures, Porton Down, UK), and U937 were purchased from ATCC (ATCC^®^ CRL-3253™).

### 2.1. Poly(allylamine)-oxadiazole (PAA-Ox5) Synthesis and Characterisation

PAA-Ox5 was synthesized as previously reported [22]. PAA was reacted in 1:0.05 molar feed with 5-(4-chlorophenyl)-1,3,4-oxadiazole-2-thiol (oxadiazole, Ox) (monomer/pendant). Briefly, PAA was dissolved in 1:1 (*v*/*v*) methanol/chloroform with stirring, followed by the addition of 3× excess of triethylamine. The reaction was stirred for 0.5 h at room temperature. To the polymer solution was added 5-(4-chlorophenyl)-1,3,4-oxadiazole-2-thiol, dissolved in 1:1 (*v*/*v*) methanol/chloroform, in a drop-wise manner over 0.5 h. The reaction was stirred for a further 18 h at 37 °C. After this time, the solvent was evaporated and the polymer residue washed 3× with diethyl ether before air drying overnight. The resultant residue was dissolved in deionised water and dialysed (12–14 kDa pore size) against deionised water for 24 h. The resultant solution was freeze-dried. Polymer structure was characterised using proton nuclear magnetic spectroscopy (^1^H NMR, 400 MHz at 25 °C) in deuterated methanol (Bruker, MA, USA), attenuated total reflectance-fourier transform infrared spectroscopy (ATR-FTIR) (Nicolette IS50, Thermo-Fisher, Loughborough, UK), and elemental analysis on a Perkin Elmer series 2 elemental analyser (Perkin Elmer, MA, USA).

### 2.2. HNP Synthesis and Characterisation

HNP synthesis has been well reported previously [23,24,25]. Briefly, iron oxide (Fe_3_O_4_) nanoparticles were prepared through a precipitation reaction and stabilised with poly(ethylenimine) (MW 200,000). Iterative gold coating was achieved by reduction of tetrachloroauric acid onto gold seeding on the HNP surface. Metal content was determined using inductively coupled plasma–optical emission spectroscopy (ICP-OES). The concentration of HNPs used for all experiments indicates the concentration of Fe. Maximum ultraviolet (UV) absorbance (λ_max_) of HNPs was measured using a UV-2600 ultraviolet visible (UV-Vis) spectrophotometer with an ISR-2600 Plus Integrated sphere (Shimadzu, Kyoto, Japan). The samples were scanned in aqueous solution, inside quartz cuvettes between 400 and 800 nm. The hydrodynamic diameter and polydispersity index were estimated using photon correlation spectroscopy (PCS) (Zetasizer Nano-ZS, Malvern Instruments, Malvern, UK). All measurements were carried out at 25 °C (*n =* 3). Zeta potential measurements were carried out to determine surface charge using the same instrument. Transmission electron microscopy (TEM) was used to visualise the morphology and diameter of particles on formvar-coated copper grids using a JEOL JEM-1230 microscope with ANAlysis software (JEOL, Tokyo, Japan). 

### 2.3. PAA-Ox5-HNP Conjugation

A 10 mL solution of 5 mg/mL PAA-Ox5 was prepared with deionised water as the diluent. The polymer solution was probe sonicated for 10 min. To this solution, 500 µL of HNPs were added and the mixture was probe sonicated for a further 10 min. 

### 2.4. Characterisation of PAA-Ox5 and PAA-Ox5-HNP Nano-Aggregates

Nano-aggregates were formed in aqueous solution after probe sonication. Solutions were filtered using 0.45 µm syringe filters prior to analysis. The critical aggregation concentration (CAC) was determined using a torsion balance. The surface tensions of polymer solutions were measured at 25 °C using a torsion balance (OS, White Electrical Instrument Co., London, UK). The platinum ring and platform were cleaned with ethanol and doubly distilled water prior to analysis of each sample. The measurement was conducted in triplicate for each polymer solution to obtain an average value. The surface tension of deionised water was determined between each concentration to ensure no cross contamination of samples had occurred. Photon correlation spectroscopy, zeta potential measurement, TEM imaging, and magnetic characterisation was carried out as previously described. 

### 2.5. Attachment of Cytochrome C onto the HNPs in the PAA-Ox5-HNP

PAA-Ox5-HNP (10 mL, 5 mg/mL) was prepared in deionised water, 500 µL of cytochrome C (at the IC_10_ of the cytochrome C/0.012 mg/mL, determined previously [14]) was added and the solution was probe sonicated for 10 min, forming PAA-Ox5-HNP-c. The particles were magnetically separated from the solution and the amount of attached cytochrome C was quantified indirectly by measuring levels of non-attached protein. Quantification was carried out using UV-Vis spectrophotometry (ISR-2600 Shimadzu, Duisburg, Germany) at 410 nm.

### 2.6. Paclitaxel (PTX) Loading and Release of Nano-Aggregates

PTX (25 mg) was loaded into PAA-Ox5-HNP-c nano-aggregates using probe sonication with a drug/polymer feed ratio (1:1) for 10 min. The resultant solution was filtered to remove unsolubilised PTX molecules using a 0.45 μm syringe filter. PTX concentration was determined using reverse phase high performance liquid chromatography (HPLC) with a mobile phase consisting of 55:45 (*v*:*v*) water/acetonitrile using a Perkin Elmer Flexar LC Auto sampler (Perkin Elmer, Waltham, MA, USA) and a flow rate of 1 mL/min, and analysed using UV detection at 227 nm. All measurements were performed at room temperature and PTX encapsulation concentration was calculated in respect to the PTX standard curve. PTX formulation was analysed using photon correlation spectroscopy, zeta potential, and TEM imaging. The formulation was also freeze-dried for NMR and ATR–FTIR spectra measurements. PAA-Ox5-HNPc+PTX final formulation was stirred at room temperature in water for 24 h. At set time points, an external magnetic field was applied and HNPs were collected, the supernatant solution was sampled and analysed for PTX, as previously described. The samples were carried out in triplicate and compared to a calibration curve.

### 2.7. Biological Characterisation of Nano-Aggregates and Formulations

HepG2, Huh-7D12, SK-hep-1, and U937 cells were grown as a monolayer in Dulbecco’s Modified Eagle’s Medium (DMEM) culture media (HepG-2 & Huh-7D12), Eagle’s Minimum Essential Medium (EMEM) media for SK-hep-1, and Roswell Park Memorial Institute (RPMI) media for U937. All media was supplemented with 10% FBS (fetal bovine serum) and 1% penicillin streptomycin. The cells were grown in a humidified incubator at 5% CO_2_ and 37 °C. The U937 cells were differentiated with 0.02% phorbol-12-myristate 13-acetate (PMA) (50 μg/mL in phosphate buffered saline (PBS)) in order to transform them into macrophage-like cells before testing.

The level of cytotoxicity of the PTX formulation was determined via the 3-[4,5-dimethylthiazol-2-yl]-2,5-diphenyltetrazolium bromide (MTT) assay on the four cell lines over 24 h. Briefly, the cells were seeded into 96-well plates (15,000 cells/well) and incubated with increasing PTX concentration (0–1 nM). The plates were incubated for 24 h before the solutions were removed and cells washed with fresh media. MTT solution (50 µL, 5 mg/mL) was added to the wells and the plate was incubated (37 °C with 5 Männedorf, Switzerland). The percentage viability was calculated relative to the positive and negative controls (media and Triton-X (1:5 phosphate buffered saline (PBS), respectively). Cytotoxicity assays for free PTX and PAA-Ox5-HNP-c incorporated PTX were carried out. 

Drug uptake studies were carried out after 4 h incubation of the PTX formulation. The cells were seeded in a six-well plate (50,000 cells/well) and incubated with 50 µg/mL PTX (in formulation compared with free drug). The plates were incubated for 4 h at 37 °C and 5% CO_2_ before the media was removed and the cells washed three times with phosphate buffered saline (PBS). The cells were trypsinised and resuspended in 1 mL media. The cells were counted on a Countess^®^ automated cell counter (Thermofisher, Loughborough, UK), 100,000 cells were transferred into an Eppendorf tube and centrifuged at 500 rpm for 5 min. The cell pellet was resuspended in deionised water and vortexed for 30 s. The total intracellular concentration was measured using reverse phase HPLC with UV detection at 227 nm, using HPLC (Perkin Elmer Flexar LC Autosampler) as previously described. Total drug concentration was calculated per cell in respect to calibration curves.

All statistical analyses were carried out using *t*-test analysis within the Microsoft Excel^®^ software package (Microscoft, Redmond, WA, USA).

## 3. Results

### 3.1. Synthesis and Characterisation of PAA-Ox5

**Figure 2:**
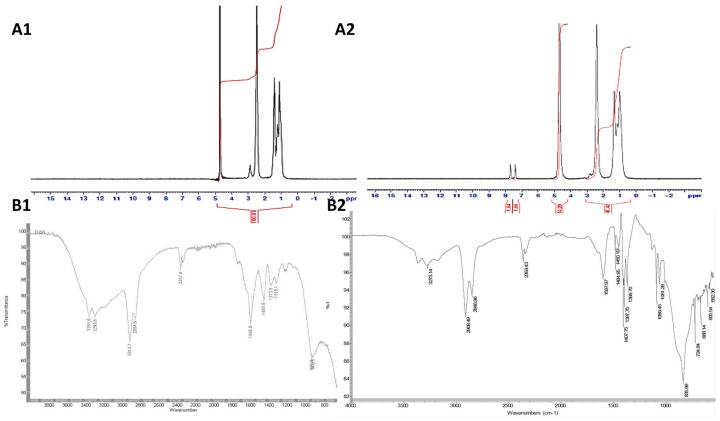
Chemical characterisation of (**A1**) PAA using proton nuclear magnetic resonance (^1^H NMR) spectroscopy, (**A2**) PAA-Ox5 using proton nuclear magnetic resonance (^1^H NMR) spectroscopy, (**B1**) PAA using attenuated total reflectance–fourier transform infrared (FTIR-ATR) spectroscopy and (**B2**) PAA-Ox5 using attenuated total reflectance–fourier transform infrared (FTIR-ATR) spectroscopy.

The fabrication of PAA-Ox5 was performed via a simple substitution reaction of the primary amine in the parent PAA structure and the chloride functional group of the oxadiazole compound. ^1^H NMR spectroscopy confirmed that the comb polymer synthesis was successful (Figure 2A). Peaks were observed at δ 0.75 ppm, δ 1.40 ppm, δ 1.50 ppm, δ 2.50 ppm and δ 3.00 ppm, which are because of the CH_2_ and CH groups in the parent PAA backbone (Figure 2A1), respectively. Additional peaks were observed at δ 7.25–7.75 ppm after Ox5 conjugation onto the polymer backbone (Figure 2A2). These were a result of the aromatic CH groups present in the oxadiazole pendant group. The proton on the –SH group of the oxadiazole pendant was not detected, which is likely to be a result of rapid exchange between the thiol and the D_2_O solvent, hence masking the signal.

FTIR analysis of the PAA-Ox5 amphiphile showed characteristic peaks observed at 3273 cm^−1^ and 2846 cm^−1^, which are attributed to the N–H stretching and C–H stretching, respectively, in the PAA backbone (Figure 2B1). After attachment of the Ox5 onto the PAA backbone, an additional peak was observed at 1597 cm^−1^ as a result the bending and stretching vibrations of the C=C in the aromatic ring (Figure 2B2). The surface tension for the PAA amphiphiles, both with and without incorporation of HNPs, was determined.

### 3.2. Synthesis and Characterisation HNPs

**Figure 3:**
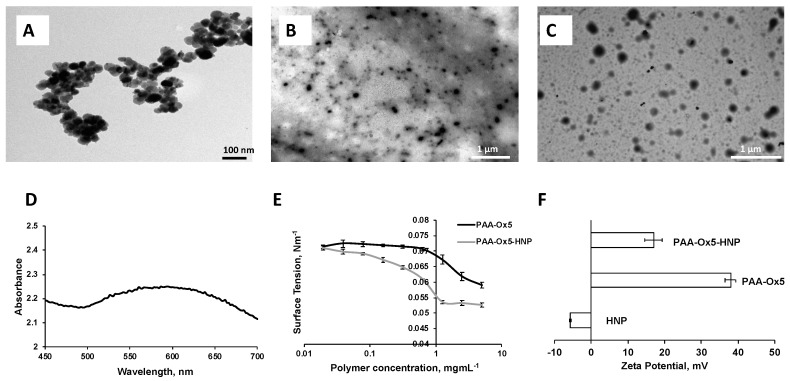
Physical characterisation. Transmission electron microscopy (TEM) images of (**A**) HNPs, (**B**) PAA-Ox5, and (**C**) PAA-Ox5-HNP. (**D**) Ultraviolet visible (UV-Vis) spectroscopy of HNPs, (**E**) surface tension measurements of amphiphiles, and (**F**) zeta potential measurement of samples in deionised water (*n =* 3, ± standard deviation—SD).

The metal (Fe and Au) composition of the HNPs was determined using ICP-OES. The data showed that 2.09 mg/mL of iron and 1.16 mg/mL of gold were present, giving a 2:1 Fe/Au ratio (Table 1). The particle size, as observed on TEM, was approximately 40 nm and the particulates were spherical in morphology (Figure 3A). UV-Vis spectroscopy showed a lambda max value of approximately 600 nm (Figure 3D), confirming gold coating had been achieved.

### 3.3. Characterisation of the Nano-Aggregates

As polymer concentration increases in the aqueous environment a change in surface tension of the aqueous media is observed. This is a result of the surface layer becoming saturated with amphiphile, forcing it into the bulk solution and hence initiating aggregation. The critical aggregation concentration (CAC) is a measure of how stable the aggregates are in solution and gives an indication of whether they will undergo breakdown after dilution. The surface tension showed that the CAC for the PAA-Ox5 was 0.313 mg/mL, whereas the HNP containing amphiphile was 0.039 mg/mL (Figure 3E). This suggested that the aggregates formed at lower concentrations when the magnetic HNPs were incorporated into the structure. The reason for this is not known, however it may be possible that the magnetic attractive forces between the HNPs result in stronger attractive forces than the intermolecular hydrophobic–hydrophobic forces required for aggregation. Hence, these drive forward aggregation at lower concentrations.

TEM imaging showed the PAA-Ox5 micelles structure of approximately 75 nm in diameter (Figure 3B). This was much smaller after HNP inclusion into the polymer structure. It was observed that the aggregated formed from PAA-Ox5-HNP (150 nm, Figure 3C) exhibited a dense core (due to the HNPs) with an irregular outer polymer shell with hindered ability to form compact aggregates when compared with the PAA-Ox5. Photon correlation spectroscopy measured particulates of 82 nm for PAA-Ox5 and 186 nm for PAA-Ox5-HNP (Table 1). The slight variance is because of the difference between measurement techniques, that is one is carried out on a dry sample and one in solution, with the hydrophilic corona also being measured. The poly dispersity index of the PAA-Ox5 was found to be 0.55, which does not indicate that particles are particularly mono-dispersed (Table 1). After addition of the HNP into the polymer structure, a decrease in polydispersity index (PDI) (0.137) was observed, indicating that the aggregates formed were much more mono-dispersed; a desirable quality for drug delivery systems.

The PAA-Ox5 zeta potential was measured to be +38 mV. This highly cationic value is because of the presence of amine groups in the PAA backbone (Figure 3F). After HNP conjugation, this value reduced to +17 mV. The reason for this is not known; as we postulate that the HNPs sit inside the aggregate core, there should be no change to net charge. Perhaps some of the particles appear close to the shell surface, influencing the surface properties of the aggregates.

### 3.4. Quantification of Cytochrome C onto the HNPs in the PAA-Ox5-HNP

The amount of cytochrome C attached to the HNPs inside the PAA-Ox5-HNP aggregates was measured using UV-Vis spectroscopy at 410 nm. The concentration of the pro-apoptotic protein was measured to have 100% attached into the formulation. This finding showed that, despite the nano-aggregate forming the hydrophobic core-shell structure, the dative covalent binding between the gold of the HNP surface and cysteine in the cytochrome C structure was great enough to pull the hydrophilic protein inside the self-assembly.

### 3.5. PTX Loading and Release of Nano-Aggregates

**Figure 4:**
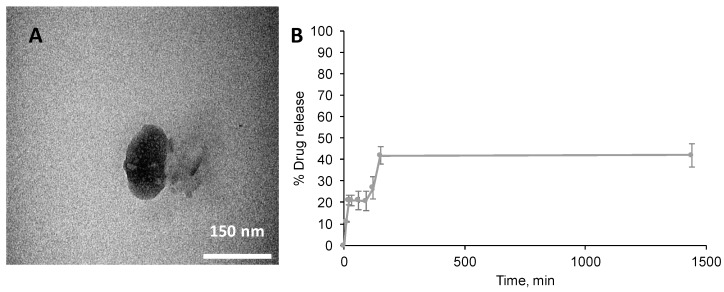
Properties of PAA-Ox5-HNPc+paclitaxel (PTX) loaded formulations. (**A**) TEM image of nano-aggregate and (**B**) drug release profile over 24 h carried out at room temperature (*n =* 3, ±SD).

PTX was incorporated into the nano-aggregates via insertion into the hydrophobic core. Interestingly, the PAA-Ox5-HNP-c nanoparticles resulted in higher levels of encapsulation (0.684 mg/mL) when compared with the PAA-Ox5 (0.598 mg/mL), as observed in Table 1. This is probably because of the less compact, larger aggregate formed by the PAA-Ox5-HNP, which possessed increased capacity for the bulky PTX molecules within their hydrophobic domain. The aqueous solubility of PTX was increased 700-fold for PAA-Ox5-HNP-c aggregates when compared with its intrinsic aqueous solubility. The final formulation of PAA-Ox5-HNPc+PTX resulted in a nano-aggregate measuring approximately 175 nm using TEM (Figure 4A), and 256 nm using photon correlation spectroscopy (Table 1).

Drug release from PAA-Ox5-HNPc+PTX was measured over 24 h (Figure 4B). The data showed that a preliminary release of 20% PTX was observed after 20 min, this increased to approximately 40% after 150 min, no further release was detected over the time-frame tested. Therefore, it is presumed that this platform may be useful as a delivery system that provides an initial bolus dosage followed by a steady maintenance dose. More work is required in order to investigate this potential under more realistic physiological conditions.

### 3.6. Biological Characterisation of Nano-Aggregates and Formulations

**Figure 5:**
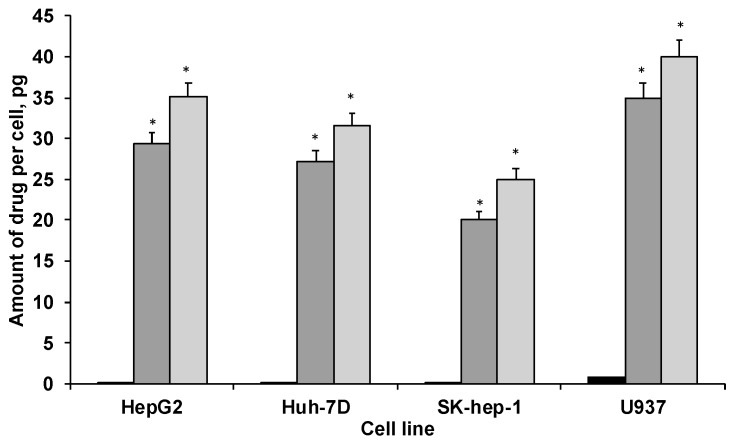
Cellular uptake of PTX in HepG2, Huh-7D12, SK-hep-1 and U937 cells after 4 h exposure to drug alone and in formulation (*n =* 3, ±SD). ■ PTX, 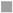 PAA-Ox5+PTX, 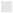 PAA-Ox5-HNPc+PTX. * denotes significance compared with free drug, *p* < 0.001.

Cellular uptake of PTX, both in native form and in the PAA-Ox5-HNP-c formulation, was measured in HepG2, Huh-7D12, SK-hep-1 and U937 cells after 4 h, using ICP-OES. The data (Figure 5) showed that drug internalisation levels consistently and significantly increased after encapsulation inside the polymer amphiphile when compared with the native form across all four cell lines. This is likely because of the nanoparticles undergoing endocytosis, an extensively reported mechanism for cellular internalisation of nanomedicines. This process results in much more rapid cellular internalisation, as well as allowing greater quantities to become incorporated into the intracellular environments when compared with the conventional mechanisms for drug uptake, such as permeation through tight junctions, passive transport, etc. Interestingly, the PAA-Ox5 containing PTX consistently showed significantly reduced drug internalisation levels when compared with the PAA-Ox5-HNP. As these complex structures form polymeric nano-aggregates with the HNPs inside the hydrophobic core, it is expected that there should be little difference in their surface properties, resulting in different internalisation levels. However, the zeta potential data did show a reduction in surface charge from +38 mV to +17 mV (Figure 3F) upon HNP conjugation. This finding, coupled with the increased drug loading levels of PAA-Ox5-HNP (Table 1), possibly account for the increased PTX levels inside all cell lines tested.

The differentiated U937 cells possessed elevated intracellular PTX concentrations across the board. This may be because of the nature of these ‘macrophage-like’ cells, internalizing the drug/formulations in line with their role within biological systems—to seek and destroy.

**Figure 6:**
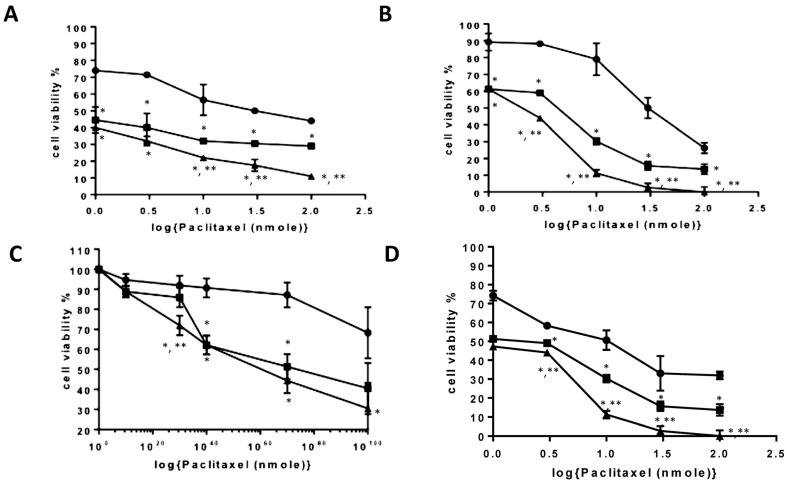
Cytotoxicity of formulations in (**A**) hepatocellular carcinoma cell line (HepG2), (**B**) Huh-7D12, (**C**) SK-hep-1 and (**D**) U937 cells after 72 h exposure to PTX alone and after encapsulation inside amphiphiles (*n =* 3, ±SD). ● PTX, ■ PAA-Ox5+PTX, ▲ PAA-Ox5-HNPc+PTX. * denotes significance compared to free drug and ** denotes significance compared to PAA-Ox5+PTX, *p* < 0.01.

The cytotoxicity of the PTX-loaded PAA-Ox5-HNP-c amphiphiles on liver cancer cell lines (HepG2, Huh-7D12, SK-hep-1, and U937) was detected via MTT assay (Figure 6). Serial dilutions of PTX alone and in combination with the PAA-Ox5-HNP-c formulation for 24 h, 48 h, and 72 h.

After 24 h incubation with HepG2 cells, the IC_50_ was determined to be 50 nM and 40 nM in the PTX formulations of PAA-Ox5 and PAA-Ox5-HNP-c, respectively (Table 2). This was a significant improvement compared with free drug, where no IC_50_ was observed in the concentration ranges tested. A similar trend was observed in the Huh-7D12 cells, whereby the PTX alone did not result in 50% loss in viability, however within the nanoparticle formulations, IC_50_ values were determined to be 53 nM and 11 nM for PTX encapsulated within PAA-Ox5 and PAA-Ox5-HNP-c, respectively. The SK-hep-1 cell line is a notoriously drug resistant cell line, and in keeping with previous findings, there was no observed IC_50_ across the concentrations tested for all samples. In general, increasing the exposure time resulted in a reduction of the IC_50_ value, as presumably more of the drug entered the cells.

After 72 h exposure in the HepG2 cells, a 31-fold and 43-fold decrease in IC_50_ of PTX formulated in the PAA-Ox5 and PAA-Ox5-HNP-c formulation, respectively, compared with free drug was observed (Figure 6A), with similar trends observed in the Huh-7D12 cells (Figure 6B). Again, the SK-hep-1 cell line proved the most resistant to treatment, with no IC_50_ being observed after 72 h PTX drug treatment. However, significant PTX formulations of PAA-Ox5 and PAA-Ox5-HNP-c resulted in cell viability reduction, with IC_50_ values observed at 73.32 nM and 57.39 nM, respectively (Figure 6C).

Interestingly, across the board, the differentiated U937 cells exhibited the greatest reduction of cell viability after exposure to the drug/formulations (Figure 6D). This is not surprising given the nature of these cells and their rapid internalisation processes, and does indeed match with the cellular internalisation data. Additionally, these cells are relatively small in comparison with the other cell lines, and hence, this coupled with increased PTX intracellular levels may possibly amplify the effect.

## 4. Discussion

Overall, the findings from this study show that formulation of PTX into a nano-sized preparation did increase the drug efficacy when compared with free PTX. This result is presumably because more of the drug is entering the cell, as observed in the cellular uptake studies (Figure 5). This is in agreement with other studies in the literature, whereby, after encapsulation into a polymeric nanoparticle, greater intracellular trafficking of drug compounds is observed [26,27,28]. Additionally, formulation of this practically insoluble drug renders it more clinically useful, as aqueous preparations of injectables are preferred over oil or organics because of reduced pain on administration, as well as toxic side effects from the diluents. This is a significant drive within the pharmaceutical arena in order to reformulate and repurpose existing drug entities, which may be hindered by their physicochemical properties or indeed may have failed at clinical trial because of these [29,30].

There was significant enhancement in cytotoxic effect observed using the PAA-Ox5-HNP-c when compared with the PAA-Ox5 (Figure 6). This indicates that the cytochrome C presence enhances the toxic effect of formulation, as we previously observed [13,14]. These exciting data show that combination therapy does indeed result in increased overall efficacy. As the relative cost of cytochrome C is less than for cytotoxic agents, this may serve as a feasible alternative to combining two chemotherapies. Although, in this study, the combined therapy does not result in huge levels of synergy, as when the HNP-c and PTX were co-administered as separate preparations previously, it does give promise that the concept matches the in vitro data—hence, formulation optimisation will ultimately improve efficacy [14]. The possible reason for the reduced combination efficacy when compared with the previous work may be because of the use of the cytochrome C at its own IC_50_ value, which was later diluted across the concentration ranges. It would be more valuable and possibly result in greater efficacy if this HNP-c was introduced to the cells at a fixed level with only the drug concentration varied. Further studies are underway to optimise this formulation approach and further elucidate whether the introduction of cytochrome C into the polymer renders a greater extension of cytotoxic efficacy on the liver cancer cells. These studies are vital in order to create a sufficient efficacy enhancement to warrant clinical translation. Additionally, in order to gain a full picture in terms of cellular toxicity, a non-cancerous liver cell line will also be included in our studies.

The most exciting finding from this data is that the nano-formulation results in much more rapid cellular uptake compared with PTX alone, presumably because of the initiation of endocytosis. In cancer, the tissue proliferates so rapidly that every day counts. Often for liver cancer, later diagnosis occurs due to lack of specific symptoms. Hence, any advancement on a three-day post administration effect is a success. Here, we saw IC_50_ values evident after only 24 h—we did not test before this time point, but looking at the cellular uptake levels after only 4 h, it is likely that this cytotoxic effect does also occur pre-24 h. The SK-hep-1 cell lines, which proved extremely problematic in our previous extended study [14], appeared to be much more susceptible to this single macromolecular nano-formulation when compared with administration of the PTX and HNP-c together. This is a big achievement and gives hope to overcoming the drug resistance that is already evident in liver cancer treatment.

## 5. Conclusions

This platform offers an exciting solution to overcoming the renowned difficulty in the formulation of PTX, as well as allowing for combination therapy with cytochrome C. Further formulation optimization is needed in order to fully exploit the synergistic efficacy observed in vitro and, ultimately, in vivo translation will elucidate the further potential of this platform as a feasible treatment for liver cancer.

## Figures and Tables

**Table 1:** Physical properties of particles and formulations developed (*n =* 3, ± standard deviation—SD). PTX—paclitaxel; HNP—hybrid iron oxide-gold nanoparticle.

Sample	Particle Size Using Photon Correlation Spectroscopy, nm	Poly Dispersity Index	Metal Content Fe/Au	Cytochrome C Content mg/mL	PTX Content mg/mL
HNP	948	1	2:1	-	-
PAA-Ox5	82	0.55	-	-	-
PAA-Ox5-HNP-c	186	0.137	2:1	0.012	-
PAA-Ox5+PTX	112	0.232	-	-	0.598
PAA-Ox5-HNP-c+PTX	256	0.124	2:1	0.012	0.698

**Table 2:** IC_50_ values observed over 24–72 h exposure of PTX alone and in formulation in HepG2, Huh-7D12, SK-hep-1 and U937 cell lines (*n =* 3, ±SD).

HepG2 Cell Line	PTX	PAA-Ox5+PTX	PAA-Ox5-HNP-c+PTX
24 h	-	50 ± 3.05 nM	40 ± 1.5 nM
48 h	-	1.99 ± 0.7 nM	0.98 ± 1.3 nM
72 h	32 ± 4.84 nM	1 nM	0.7 nM
**Huh-7D12 cell line**			
24 h	-	53 ± 1.52 nM	11 ± 2.5 nM
48 h	37 ± 1.05 µM	4 ± 2.61 nM	2.21 ± 1.82 nM
72 h	31 ± 1.12 µM	3 ± 2.5 nM	0.9 ± 2.1 nM
**SK-hep-1 cell line**			
24 h	-	-	-
48 h	-	-	84.27 ± 3.1 nM
72 h	-	73.32 ± 1.54 nM	57.39 ± 4.02 nM
**U937 cell line**			
24 h	-	14 ± 1.5 nM	3.6 ± 0.45 nM
48 h	39 ± 1.72 nM	11 ± 1.29 nM	2.6 ± 0.63 nM
72 h	9 ± 1 nM	1.6 ± 1.08 nM	1.2 ± 2.24 nM
